# Rational dosage regimens for cephalothin and cefazolin using pharmacokinetics and pharmacodynamics analysis in healthy horses

**DOI:** 10.1111/evj.13406

**Published:** 2021-01-21

**Authors:** Taisuke Kuroda, Yohei Minamijima, Hidekazu Niwa, Norihisa Tamura, Hiroshi Mita, Kentaro Fukuda, Masahiro Kaimachi, Yuto Suzuki, Yuki Enoki, Kazuaki Taguchi, Kazuaki Matsumoto, Pierre‐Louis Toutain, Alain Bousquet‐Melou, Yoshinori Kasashima

**Affiliations:** ^1^ Clinical Veterinary Medicine Division Equine Research Institute Japan Racing Association Shimotsuke Japan; ^2^ Drug Analysis Department Laboratory of Racing Chemistry Utsunomiya Japan; ^3^ Microbiology Division Equine Research Institute Japan Racing Association Shimotsuke Japan; ^4^ Division of Pharmacodynamics Keio University Faculty of Pharmacy Tokyo Japan; ^5^ Comparative Biomedical Sciences The Royal Veterinary College London UK; ^6^ INTHERES, Université de Toulouse, INRA ENVT Toulouse France

**Keywords:** horse, cefazolin, cephalothin, gram positive infection

## Abstract

**Background:**

First‐generation cephalosporins have good activity against gram‐positive bacteria and are extensively used in horses. There are few reports of pharmacokinetics and pharmacodynamics (PK/PD) analysis of cephalosporins in horses.

**Objective:**

To optimise the dosages of the two first‐generation cephalosporins cephalothin (CET) and cefazolin (CEZ) in horses using PK/PD concepts.

**Study design:**

Experimental study with single administration.

**Methods:**

Drug plasma concentrations following a single intravenous (i.v.) administration of 22 mg/kg bodyweight (bwt) CET in 12 horses and of 10 mg/kg bwt CEZ in six horses were measured using LC‐MS/MS. Data were modelled using a nonlinear mixed effect modelling followed by Monte Carlo simulations. Minimum inhibitory concentrations (MICs) against *Streptococcus zooepidemicus* and *Staphylococcus aureus* isolated from horses were determined by the microbroth dilution method.

**Results:**

The percentages of CET and CEZ binding to serum proteins were 19.9% ± 8.4% and 15.2% ± 8.5% respectively. For both CET and CEZ, the MIC_90_ against *S. zooepidemicus* was 0.12 mg/L and against *S. aureus* was 0.5 mg/L. For CET, to achieve a probability of target attainment (PTA) of 90% for a PK/PD target of a free serum plasma concentration exceeding the MIC_90_ for 40% of the dosing interval, an empirical CET dosage regimen of 22 mg/kg bwt q8h and 22 mg/kg bwt q4h i.v. administration were required for *S. zooepidemicus* and *S. aureus* respectively. For CEZ, the corresponding dosage regimens were 10 mg/kg bwt q12h and 10 mg/kg bwt q8h.

**Main limitations:**

Small sample size only in healthy horses.

**Conclusions:**

For CET, more frequent administration than that currently recommended (22 mg/kg bwt q6–12h) is required to empirically control *S. aureus* infection in horses. For CEZ, less frequent administration compared to the dosage regimen currently proposed (10–22 mg/kg bwt q6h) could control *S. zooepidemicus* and *S. aureus* infections in horses.

## 1 INTRODUCTION

*Streptococcus equi subsp. zooepidemicus* is a gram‐positive, β‐haemolytic coccus belonging to the Lancefield group C. It is an opportunistic pathogen for adult horses, and it can cause shipping fever and pneumonia that could be fatal.^1^, ^2^ In addition, *Staphylococcus* spp. and *Streptococcus* spp. are frequently isolated from cellulitis in horses.^3^ These gram‐positive bacteria are considered as common pathogens for respiratory and limb infection in horses. β‐Lactams, including penicillin and cephalosporins, are effective against gram‐positive bacteria. Penicillin is commonly used in horses; however, it may not be effective against staphylococci owing to the development of resistant strains.^4^, ^5^

The first‐generation cephalosporin, cefazolin (CEZ), has been used in horses^6^, ^7^ with a default recommended dosage regimen of 10–22 mg/kg bwt q6–8h in textbook and 25mg/kg bwt q6h in Clinical and Laboratory Standards Institute (CLSI) standards.^8^, ^9^ However, the recommended dose has a 2‐fold range in textbooks, and the rational dosage for each organism has not been determined. Cephalothin (CET) is also a first‐generation cephalosporin that has been previously reported in horses^10^, ^11^ and is listed in equine textbooks.^12^, ^13^ CET is no longer sold in certain countries but is still available in South America, Europe, Australia and Asia as a human drug and an off‐label prescription drug for horses. For CET, a dose of 22 mg/kg bodyweight (bwt) q6–12h is routinely but empirically used in horses in Japan,^14^ but this regimen has not been rationally determined using pharmacokinetics/pharmacodynamics (PK/PD) analysis. Because of increasing bacterial resistance against third‐generation cephalosporins used in livestock and companion animals and of its consequent risk to humans,^15^, ^16^ the dosing schedules for these two cephalosporins which can be used as first‐line antimicrobials in horses must be updated. In this report, PK/PD analysis was conducted based on the pharmacokinetics of CET and CEZ and on their minimum inhibitory concentrations (MICs) against bacteria isolated from horses to optimise their dosage regimen.

## 2 MATERIALS AND METHODS

For the CET study, 12 healthy 3‐5‐year‐old experimental Thoroughbred horses (six stallions and six mares) with bodyweights (bwts) of 410‐530 kg were used. For the CEZ study, six healthy 3‐5‐year‐old horses (four stallions and two mares) with bodyweights of 425‐530 kg bwt that were also enrolled in the CET study were used. Horses were kept in individual stalls during the experiments and had ad libitum access to grass, hay and water.

For the six horses in both the CET and CEZ studies, a 2 × 2 crossover design was carried out with a 2 weeks washout period; the horses were randomly allocated to the two sequences. The doses of the cephalosporins, 22 mg/kg bwt CET and 10 mg/kg bwt CEZ, were determined based on previous reports.^6^, ^11^ CET (Coaxin injection 1 g) (Chemix Inc) was dissolved in 50 mL sterile physiological saline and CEZ (Cefazolin sodium injection 1 g) (Fujita Pharmaceutical Company) was dissolved in 30 mL sterile physiological saline for intravenous (i.v.) administration into the right jugular vein by a short bolus infusion (<30 seconds). The CET formulation was approved for humans, and that of CEZ for animals was approved in Japan.

Blood samples were collected at time 0 (prior to administration) and at 5, 10, 20, 30 and 45 minute and 1, 2, 3, 4, 6, 8 and 12 hour after administration. All blood samples were taken from the left jugular vein using a 14G catheter (Becton Dickinson Company), and 10 mL blood samples were collected in heparinised vacuum blood collection tubes (Terumo). The samples were immediately centrifuged at 1,500 g for 10 minute, and the separated plasma samples were stored at −20°C until analysis.

### 2.1 Determination of plasma concentrations

Concentrations of CET, its active metabolite, deacetylcephalothin (DCET) and CEZ were measured. Quality control samples for calibration of the plasma analysis were prepared by adding standard CET (Toronto Research Chemicals Inc.), DCET (Toronto Research Chemicals Inc.) and CEZ (FUJIFILM Wako Pure Chemical Corporation) to blank horse plasma. To 20 μL of plasma was added 400 μL of acetonitrile and 20 μL of 1 μg/mL oxacillin sodium monohydrate (AdooQ BioScience) as an internal standard. The sample was incubated for 5 min at room temperature and centrifuged at 10 000 g for 5 min. Fifty microlitres of supernatant was transferred to a new vial and diluted with 250 μL of water. Five microlitres of the sample was injected into a liquid chromatography system (Nexera X2) (Shimadzu Corporation) connected to a mass spectrometer (QTRAP4500) (SCIEX Corporation). High‐performance liquid chromatography separation was performed on the column (ACQUITY UPLC BEH, 100 mm × 2.1 mm, 1.7 μm) (Waters Corporation) with a mixture of formic acid (0.1 vol%) and acetonitrile as the mobile phase. The final calibration curve had a coefficient of correlation (R^2^) >0.995 over the concentration range of 0.1–300.0 µg/mL for CET and DCET and the range of 0.03‐100.0 µg/mL for CEZ. The lower limit of quantitation (LOQ) was 0.1 µg/mL for CET and DCET and 0.03 µg/mL for CEZ. The recovery ratios in quality control samples were determined at concentrations of 0.3, 5 and 240 µg/mL for CET and DCET and 0.09, 2 and 80 µg/mL for CEZ (five replicates each). Interday and intraday precision were assessed in quality control samples at concentrations of 0.1, 0.3, 5 and 240 µg/mL for CET and DCET and of 0.03, 0.09, 2 and 80 µg/mL for CEZ (five replicates each), and their coefficients of variation were <10% except for that of 0.03 µg/mL of CEZ, which was 14.8%. Accuracies for CET, DCET and CEZ were between 97.6% and 103.4%, 90% and 105.7%, and 86% and 103.2% respectively.

### 2.2 Protein binding

The ultrafiltration method was used to separate free and bound drug for CET, DCET and CEZ; 200 µl samples were placed in a filter (Pierce™ Protein Concentrators PES, 10K MWCO) (Thermo Fisher Scientific) and centrifuged at 15 000 g for 5 minute at room temperature. Then the free drug concentration following ultrafiltration and the total drug concentration in samples not subjected to ultrafiltration were quantified using the same assay method as previously described. The plasma samples for assay were collected at 1, 2 and 3 hour after administration. The extent of protein binding and the free fraction were calculated by comparing the free and total drug concentrations. The average free fraction was used for the simulation of free plasma concentrations of CET and CEZ.

### 2.3 Pharmacokinetic data analysis

Plasma pharmacokinetic analyses were conducted using a nonlinear mixed effect (NLME) model using commercially available software (Phoenix WinNonlin version 6.4) (Certara, Princeton). A three‐compartment structural model was selected based on the likelihood ratio test and the Akaike information criterion. The model was parametrised in terms of clearance and volume of distribution. The estimated parameters were the central (V1) and two peripheral (V2, V3) volumes of distribution, plasma clearance (CL) and the inter‐compartmental distribution clearances (CL2, CL3).

In a population model, the statistical model describing the inter‐animal variability is included in the structural model. The interindividual variation for a given parameter was described using an exponential model of the form:

(1) $$\theta_{parameter\_i}=\theta_{tv\_parameter}\cdot EXP\left(\eta_{i}\right)$$

where θ*_parameter_i_* is the value of theta for a given parameter in the ith animal, θ_tv_parameter_ is the typical population value of parameters and ηi (eta_i_) is the deviation associated with the ith animal from the corresponding theta population value. An exponential model was selected because the estimated theta parameters must be positive and their distributions are generally right‐skewed. Thus, variability between horses was estimated from their individual etas; the distribution of the etas was assumed normal with a mean of 0 and a variance ω^2^.

To report the interindividual variability as a coefficient of variation, Equation (2) was used for conversion of the variance terms (ω^2^) into a coefficient of variation (CV%).

(2) $$CV\left(\%\right)=100\times \sqrt{\exp\left(\omega^{2}\right)‐1}$$

Shrinkage of the random effects (eta) toward the means was described as follows:

(3) $$shrinkage=1‐\frac{var\left(\eta_{r}\right)}{\omega^{2}}$$

where *var(η_r_)* is the variance of the random effects. When the shrinkage for eta was >0.3, it was considered that the data were not able to robustly estimate this random component. It was impossible to estimate this between‐subject variability for all structural parameters (non‐identifiability) and a random component was added only for V, CL, and V2 for CET and V, CL and V3 for CEZ. The residual model was an additive plus a multiplicative (proportional) model of the form.

(4) $$C\left(t\right)=f\left(\theta ,Time\right)\times \left(1+\varepsilon_{1}\right)+\varepsilon_{2}$$

with ε1, the multiplicative error term having a mean of 0 and a variance noted σ1.

$$\varepsilon 1\approx N\left(0,\sigma 1^{2}\right)$$

and ε2, the additive error term having a mean of 0 and a variance noted σ2.

$$\varepsilon 2\approx N\left(0,\sigma 2^{2}\right)$$

The additive sigma was reported as its standard deviation noted with the same units as plasma concentration (µg/mL) and the multiplicative sigma was reported as coefficient of variation. For the present fitting, the precision of the parameters was estimated using the bootstrap tool (n = 50 replicates).

Using the developed model and the free fraction, Monte Carlo Simulations (MCS) were used to generate free plasma concentrations in a population of 5000 horses using individual predictions, or IPRED (eta was as estimated), corresponding to different dosage regimen scenarios. For both antimicrobials, simulation was carried out for two dose levels and at 5 interval patterns. Simulated doses were the study dose and double that dose (22 and 44 mg/kg bwt for CET and 10 and 20 mg/kg bwt for CEZ). We calculated for the 5000 curves and the time during which the free plasma concentration exceeded the MIC for 40% of the dosing interval at Day 3 after the first administration, when the steady state was achieved. We then derived the corresponding probability of target attainment (PTA) of 90%.

### 2.4 Minimum inhibitory concentrations

The MICs of CET, DCET, and CEZ were obtained using customised commercial panels (Eiken Chemical Co., Ltd.) against 98 strains of *S. zooepidemicus*, 51 strains of *Staphylococcus aureus* (without methicillin‐resistant *S. aureus*; MRSA), 54 strains of *Escherichia coli*, and 26 strains of *Klebsiella* spp. isolated from infected horses according to the CLSI standards.^17^ The horses studied were training Thoroughbred racing horses, located in two training facilities (Ritto and Miho training centres) and were sampled for infectious diseases including pneumonia and cellulitis. The MICs in the test were in the range of 0.03‐4.0 mg/L. MRSA were excluded in this study because MICs of first‐generation cephalosporins against these MRSA strains were extremely high, cephalosporins being considered ineffective.^18^

## 3 RESULTS

**FIGURE 1:**
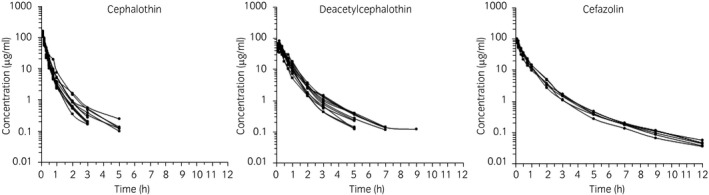
Semilogarithmic spaghetti plots of the disposition curves of cephalothin and deacetylcephalothin after a single dose administration of 22 mg/kg bwt cephalothin in 12 horses and of cefazolin after a single administration of 10 mg/kg bwt cefazolin in six horses

**FIGURE 2:**
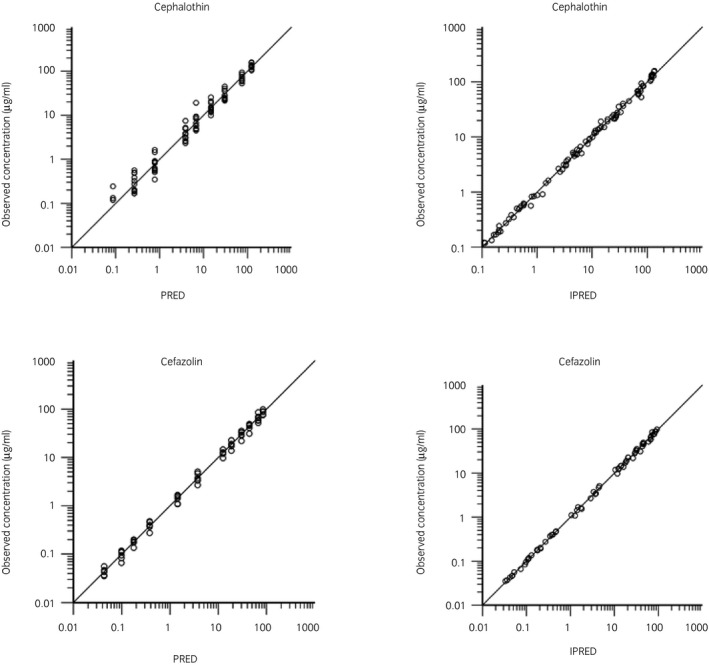
Logarithmic plots of observed cephalothin (top) and cefazolin (bottom) plasma concentrations vs population predictions (PRED) (left plots) and individual predictions (IPRED) (right plots)

**FIGURE 3:**
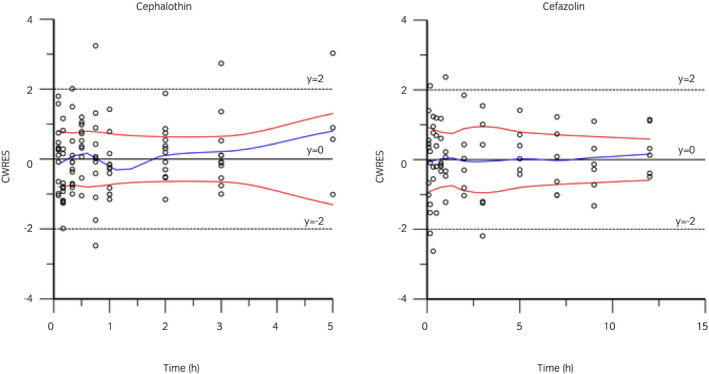
Conditional weighted residuals (CWRES) vs. time plot for cephalothin (left) and cefazolin (right). Values of CWRES should be approximately N (0, 1) and hence concentrated between y = −2 and y = +2. Inspection of the figure indicates that data were evenly distributed about zero and that the trends (as given by the blue line and the red line, its negative reflection) did not show any fanning, indicating no bias in the structural model

**FIGURE 4:**
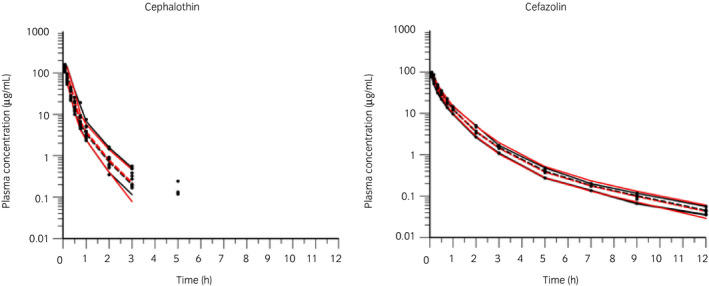
Visual Predictive Check of a single dose of 22 mg/kg bwt cephalothin (left) and 10 mg/kg bwt cefazolin (right). The observed and predicted 10th and 90th percentiles are shown in solid red and black lines respectively. The observed and predicted 50th percentiles (median) are shown in red and black broken lines respectively. Black dots are individual raw data

Semilogarithmic plots of the disposition curves of both antimicrobials and DCET in each horse are depicted in Figure 1. Logarithmic plots of the observed drug plasma concentrations vs. population predictions (PRED) and IPRED are shown in Figure 2. Data were evenly distributed about the line of identity, indicating no major bias in the population component of the model. The plot of conditional weighted residuals vs. time indicated that residuals were randomly scattered around zero with no systematic trend, supporting the selection of the residual error model for both antimicrobials (Figure 3). Bootstrap estimates of typical values of the primary structural parameters of the model (thetas), the secondary parameters and their associated coefficients of variation as a measure of the precision of their estimation are given in Table 1. Visual Predictive Check can ensure that simulated data are consistent with observed data (Figure 4). The MIC distributions of CET, DCET and CEZ are indicated in Table 2. For both CET and CEZ, the MIC_90_ against *S. zooepidemicus* was 0.12 mg/L and against *S. aureus* was 0.5 mg/L. For CET, the MIC_90_ of *E. coli* and *Klebsiella* spp. were both >4.0 mg/L. For CEZ, the MIC_90_ of *E. coli* was 2.0 mg/L and of *Klebsiella* spp. was 4.0 mg/L.

**TABLE 1:** Population primary parameters of CET and CEZ in horses with a 3‐compartment model (bootstrap estimates of median, CV%, 2.5% and 97.5% percentiles)

	Units	Median	CV%	2.50%	97.50%
CET Primary structural Parameters					
tvV	L/kg	0.103	7.01	0.086	0.113
tvV2	L/kg	0.043	14.31	0.030	0.052
tvV3	L/kg	0.045	122.74	0.032	0.283
tvCL	L/kg/h	0.551	3.64	0.509	0.582
tvCL2	L/kg/h	0.110	16.94	0.091	0.152
tvCL3	L/kg/h	0.020	23.30	0.016	0.032
tvCMultStdev (residual, proportional)	Scalar	0.126	10.63	0.146	0.099
stdev0 (residual, additive)	µg/L	0.000017	64.72	0.000010	0.000030
CET Secondary parameters					
Half_life_alpha	h	0.097	9.83	0.071	0.107
Half_life_Beta	h	0.352	16.90	0.229	0.433
Half_life_Gamma	h	1.656	82.16	0.969	7.258
Vss (steady‐state volume of distribution)	L/kg	0.191	40.62	1.512	4.224
MRT (Mean residence time (IV))	h	0.348	40.81	0.292	0.807
CEZ Primary structural Parameters					
tvV	L/kg	0.094	4.41	0.086	0.101
tvV2	L/kg	0.034	9.38	0.029	0.040
tvV3	L/kg	0.040	8.35	0.034	0.046
tvCL	L/kg/h	0.195	4.25	0.182	0.212
tvCL2	L/kg/h	0.067	21.99	0.050	0.107
tvCL3	L/kg/h	0.012	7.43	0.010	0.013
tvCMultStdev (residual, proportional)	Scalar	0.088	9.21	0.102	0.073
stdev0 (residual, additive)	µg/L	0.0000012	27.20	0.0000006	0.0000018
CEZ Secondary parameters					
Half_life_alpha	H	0.183	11.05	0.144	0.220
Half_life_Beta	H	0.579	6.16	0.538	0.679
Half_life_Gamma	H	2.567	2.44	2.448	2.654
Vss (steady‐state volume of distribution)	L/kg	0.169	4.06	0.157	0.181
MRT (Mean residence time (IV))	H	0.855	1.80	0.830	0.886

The primary estimated parameters were the volume of distribution of the central compartment (V1), the volume of distribution of the peripheral compartments (V2, V3), the plasma clearance (CL) and the distribution clearances (CL2, CL3). CMultStdev corresponds to the proportional component of the residual error and stdev0 is the additive component of the residual. The estimated fixed parameters were reported as their typical values (tv) with their CV% and their confidence interval that is a measure of the precision of their estimation. Secondary parameters are the half‐life of the different phases, the steady‐state volume of distribution (Vss) and the mean residence time (MRT).

**TABLE 2:** MIC distribution of CET, DCET and CEZ against 98 strains of *Streptococcus zooepidemicus*, 51 strains of *Staphylococcus aureus*, 54 strains of *Escherichia coli* and 26 strains of *Klebsiella* spp

Antimicrobials	Bacteria	MIC (mg/L)								
		<0.03	0.06	0.12	0.25	0.5	1.0	2.0	4.0	>4.0
CET	*S. zooepidemicus*	1	4	93						
	*S. aureus*		1	7	25	16	2			
	*E. coli*								14	40
	*Klebsiella* spp.							2	15	9
DCET	*S. zooepidemicus*				1	96	1			
	*S. aureus*				2	4	13	27	3	2
	*E. coli*									54
	*Klebsiella* spp.									26
CEZ	*S. zooepidemicus*	1	10	87						
	*S. aureus*			2	28	18	2	1		
	*E. coli*					2	31	18	2	1
	*Klebsiella* spp.						2	12	10	2

**FIGURE 5:**
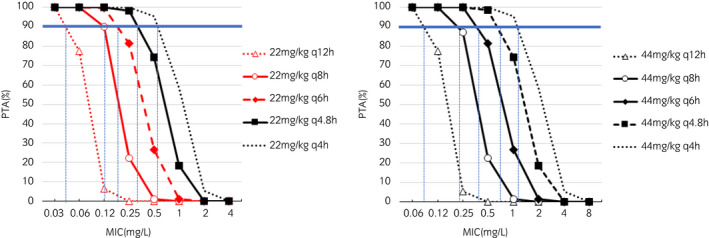
Probability of Target Attainment (PTA%) vs. MIC (µg/mL) of cephalothin for repeated administration of cephalothin 22 mg/kg bwt and 44 mg/kg bwt at different dosing intervals ranging from 4 to 12 h. The PK/PD index is the time the free plasma concentration is exceeding the MIC for 40% of the dosing interval. Values were obtained from 5000 simulated cephalothin concentrations profiles generated from the population model by Monte Carlo simulations. PTA 90% is indicated by the solid blue line, which is considered as the target to achieve, and MIC that corresponds to PTA 90% is indicated by the dotted blue line

**FIGURE 6:**
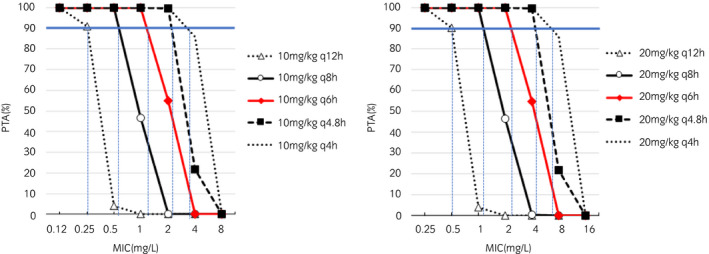
Probability of Target Attainment (PTA%) vs. MIC (µg/mL) of cefazolin for repeated administration of cefazolin 10 mg/kg bwt and 20 mg/kg bwt at different dosing intervals ranging from 4 to 12h. The PK/PD index is the time the free plasma concentration is exceeding the MIC for 40% of the dosing interval. Values were obtained from 5000 simulated cefazolin concentration profiles generated from the population model by Monte Carlo simulations. PTA 90% is indicated by the solid blue line, which is considered as the target to achieve, and MIC that corresponds to PTA 90% is indicated by the dotted blue line.

The serum protein binding percentages of CET, DCET, and CEZ were 19.9% ± 8.4%, 7.1% ± 3.9% and 15.2% ± 8.5% respectively. The PTA for the 5000 free drug concentration profiles obtained by MCS for different MICs of CET and CEZ and different regimens of the actually administered dose level are shown in Figures 5 and 6 respectively. CET 22 mg/kg bwt q8h and 22 mg/kg bwt q4h i.v. administration regimens were able to reach a PTA of 90% against the MIC_90_ of *S. zooepidemicus* and *S. aureus* respectively. For CEZ, 10 mg/kg bwt q12h and 10 mg/kg bwt q8h i.v. administration regimens were able to reach a PTA of 90% against the MIC_90_ of *S. zooepidemicus* and *S. aureus* respectively.

## 4 DISCUSSION

The pharmacokinetic parameters for CET and CEZ in this were different from those previously reported.^6^, ^7^, ^10^ These differences are correlated with those in the LOQ of analytical technics. The LOQ in previous studies was higher than that in our study. When decreasing the LOQ, a supplementary phase is often observed, leading to a decrease in plasma clearance and an increase in Vss, and a new terminal half‐life that can be significantly longer than that previously reported,^19^ as observed in the current experiment. In our study, the volumes of distribution for CET and CEZ were similar. However, CET had a higher clearance and a shorter half‐life than CEZ, thereby indicating a faster metabolic elimination. These differences were also reported in humans.^20^ CET is eliminated either directly via renal clearance or as DCET, a hepatic metabolite. Meanwhile, CEZ, which is not metabolised, is only eliminated via renal clearance.^20^

The extent of protein binding was important to establish a rational dosage regimen because only the free drug concentration is microbiologically active.^21^ For both CET and CEZ, our results indicated low protein binding, close to previously reported values in horses.^6^, ^10^ The protein binding of CET was reported as 75% and that of CEZ as 52%‐85% in humans^22^, ^23^ and of CEZ as 36.2% in dogs.^24^ These results indicate that there are wide differences in drug plasma protein binding among species, influencing the calculation of the dosage regimen in PK/PD analysis and prohibiting simple interspecific extrapolations as to the doses that should be administered to the horse. The extent of protein binding of CET in humans was decreased at high total concentrations and was considered as saturable.^23^ In the present experiment, there were no apparent differences in CET and CEZ protein binding for plasma collected at different times, and we used the average of the free fraction to make the simulations of free plasma concentration.

One report indicated MIC_90_ values of CET against *S. zooepidemicus* and coagulase‐positive *Staphylococcus* spp. were 0.06 and 0.25 mg/L respectively. These results were similar to those in our previously published study.^4^ In the current study, the MIC distributions of CET and CEZ against *S. zooepidemicus* and *S. aureus* strains were unimodal, and there was no strain with a seemingly high MIC. This result was attributed to the a priori exclusion of MRSA in this study. MRSA infections have been reported worldwide^25^ and in Japanese horses.^26^ Moreover the prevalence of penicillin‐resistant *Streptococcus pneumoniae* (PRSP) is increasing in humans, and the MIC_50_ and MIC_90_ of CEZ against PRSP were 4.0 mg/L.^27^ Because the prevalence of these resistant bacteria is increasing and they are widespread among horses, antimicrobial susceptibility testing (AST) is now necessary to ensure a prudent use of these agents with the empirical dosage regimens.

Analysis of the MIC distributions of target bacteria and PK/PD considerations can help to optimise an efficient dosage regimen or to determine MIC breakpoints for AST in humans^21^ and other animal species.^28^ Cephalosporins are considered as time‐dependent antimicrobials, for which the appropriate PK/PD index is T_>MIC_ (the time during which free plasma concentrations are above the MIC), with a typical target value of 40% of the dosing interval in humans.^29^ To establish an empirical dosage regimen (ie without resorting to an AST), the dose should cover a priori at least 90% of the horse's population for the reported MIC_90_, as can be determined by MCS of a meta‐population of 5000 horses from a population PK model.^28^, ^30^

For CEZ, our results indicate that the dose regimen may be altered depending on the MIC of the specific bacteria being treated. The CLSI reports a clinical breakpoint (CBP) for horses of <2.0 mg/L for susceptible organisms in respiratory and genital infections caused by *E. coli* and *Streptococcus spp*. Based on a dosing regimen of 25 mg/kg every 6 hour is recommended.^8^ The results of this study confirm that this dosage regimen would be effective for the control of *E. coli* which has an MIC_90_ ≤ 2.0 mg/L, and in fact doses of 20 mg/kg IV q6h may be sufficient. However, control of other Gram‐negative infections with higher MIC values would require more frequent dosing. For example, for *Klebsiella spp* (MIC_90_: 4.0 mg/L), a CEZ dose of 20 mg/kg bwt q4.8h is required. This frequent administration is not practical, and may predispose horses to the development of antimicrobial‐associated diarrhoea which has been reported following administration of both CEZ and CET, and can be severe.^31^, ^32^

Using data generated in this study, we can therefore propose a lower CBP, and therefore a lower dosage, for CEZ for treatment of *S. zooepidemicus* and *S. aureus*, with the MIC_90_ of ≤0.12 and 0.5 mg/L respectively. Using those MICs as a cut‐off, a dose of 10 mg/kg bwt IV q 8‐12h can be used to control gram‐positive infection in horses and this dosage regime is characterised by less frequent administration compared to the standard dosage regimen. In cases where concurrent infection with gram‐negative bacteria is confirmed or suspected, however, the higher dose and more frequent dosing regimen would be required. Alternatively, if the lower dose is used, combining CEZ with an aminoglycoside or fluoroquinolone, where allowed, would be recommended for gram‐negative coverage.

For CET, we found that regimens of 22 mg/kg bwt q8h and 22 mg/kg bwt q4h were appropriate for the MIC_90_ of *S. zooepidemicus* and *S. aureus* respectively. This result indicates that the current dosing regimen (CET 22 mg/kg bwt q6–8 h) could be expected to control *S. zooepidemicus* infection with a strain belonging to the wild population, but more frequent administrations would be needed to control *S. aureus*. Because q4h administration is not practical, CET continuous infusion may be efficient for *S. aureus* infection. It has been reported that, for a given total dose, continuous infusion of β‐lactams has a longer time with concentrations above the MIC and a higher cure rate than intermittent administration.^33^, ^34^ However, the current dosing regimen may have a bacteriostatic effect against *S. aureus*. In this study, the PTA was calculated for a free serum plasma concentration exceeding the MIC for 40% of the dosing interval, but a bacteriostatic effect of cephalosporins against *S. aureu*s has been reported for <30% of the dosing interval.^35^ When calculating the PTA of 30% of the dosing interval from our simulation, CET 22 mg/kg bwt q6h had a PTA of 87.4% for the MIC_90_ of *S. aureus* (not shown). In addition, DCET, which is an active metabolite of CET, was not taken into account, meaning that our dosage regimens are likely conservative. Indeed, from the raw data in this study, the time that the free DCET plasma concentration exceeded the MIC_90_ of DCET against *S. zooepidemicus* after a single dose was 3.9 ± 0.7 hour, which was similar to the 3.7 ± 0.9 hour of CET for the 12 investigated horses. An in vitro study indicated synergy or a partial synergistic effect for the combination of CET and DCET.^36^ If DCET has an additive antibacterial effect with CET in vivo, our simulations when doubling the CET dose (ie doubling the plasma CET concentration) could be relevant. Indeed, our clinical data from Japanese horses treated from 2010 to 2017 showed CET 22 mg/kg bwt q6–12h single agent administration had cure rates of 94.6% in 1240 horses with shipping fever and 94.7% in 3292 horses with limb infections (Kuroda et al., unpublished data). As well as for CEZ, combination treatment with an aminoglycoside or fluoroquinolone would be necessary for gram‐negative coverage because MIC_90_ of CET and DCET against gram‐negatives were over 4.0 mg/L.

There is currently no CBP reported for CET by CLSI or other regulatory agencies, as CET as an active pharmaceutical ingredient is not available in many countries, limiting its use in horses. Using the CLSI breakpoint for CEZ of 2 mg/L, we simulated dosing regimens for CET. This resulted in a potential CET regimen of 44 mg/kg every 4 hour which is not feasible and may result in adverse drug reactions. At least for gram‐positive bacteria, the MICs of CEZ and CET were almost the same (same in 92.5% of strains, within one dilution in 99.3% of strains in this study). Simulations run indicated that the PK/PD cutoff for MIC values ≤0.12 mg/L would be met at doses of CET of 22 mg/kg bwt IV q8h. When taking into account the active metabolite, DCET, these criteria would be met for MICs ≤0.25 mg/L.

Gram‐positive bacteria are considered common pathogens of respiratory and musculoskeletal infections in horses. Thus, β‐lactams, particularly penicillin, are used as first‐line antimicrobials. However, in some jurisdictions, procaine benzylpenicillin is a prohibited drug among racehorses. For this reason, CET is preferred as an empirical first‐line antimicrobial therapy for racehorses in Japan.^14^ Based on a PK/PD analysis, our results support the use of CET, particularly for penicillin‐resistant staphylococci. Coagulase‐positive staphylococci isolated from horses, including penicillin‐resistant strains, were sensitive to CET, erythromycin, rifampicin, lincomycin and amikacin.^5^ Of these drugs, cephalosporins are used most frequently in clinical practice because erythromycin and lincomycin can cause fatal diarrhoea in adult horses,^31^, ^37^ and the other antibiotic classes are either expensive and/or considered critical for humans.

Our study had some limitations because of the small sample size and because healthy horses that were investigated do not necessarily reflect some clinical conditions. There is the possibility that the typical plasma concentration will change in the case of severe infection as reported in horses and humans.^38^, ^39^ A population pharmacokinetic study of CEZ used prophylactically in a large population of non‐experimental dogs undergoing surgery indicated a greater variability of parameters than our result.^24^ However, our model, established with rich data sets, can now be easily updated with the sparse data collected in clinical conditions to allow coverage of situations that cannot be obtained in experimental conditions. As asserted by the VetCAST project, meta‐analysis of raw data collected in different settings (clinical or experimental) is a promising approach to establish science‐based breakpoints for the establishment of AST in veterinary species.^28^ This is allowed because the NLME modelling is an appropriate tool to merge unbalanced data obtained with analytical techniques having different performances, or in a variety of observational conditions that can be formally introduced in the modelling process.

## 5 CONCLUSION

Our study indicated that CET 22 mg/kg bwt q8h and 22 mg/kg bwt q4h i.v. administration attained therapeutic concentrations to control *S. zooepidemicus* and *S. aureus* respectively in healthy horses up to the MIC_90_ values of the wild population. When using CET for *S. aureus* infections, it must be administered more frequently than the current dosing regimen. CEZ 10 mg/kg bwt q12h and 10 mg/kg bwt q8h i.v. administrations attained therapeutic concentrations to control *S. zooepidemicus* and *S. aureus* respectively, in healthy horses. When using CEZ for gram‐positive infections, less frequent administration compared to the standard dosage regimen could be expected to control the infection. These dosage regimes can support the treatment empirically used for gram‐positive infections in horses.

## CONFLICT OF INTERESTS

No competing interests have been declared.

## 6 ETHICAL ANIMAL RESEARCH

All experiments were approved by the Animal Care and Use Committee of Equine Research Institute, Japan Racing Association.

## 7 INFORMED CONSENT

Not applicable.

## AUTHOR CONTRIBUTIONS

N. Tamura, H. Mita, K. Fukuda and Y. Kasashima contributed to study design, data collection and manuscript preparation. K. Niwa contributed to study design, MIC analysis and manuscript preparation. Y. Minamijima, M. Kaimachi, Y. Suzuki, Y. Enoki, K. Taguchi and K. Matsumoto contributed to plasma concentration analysis, pharmacokinetic analysis and manuscript preparation. P.L. Toutain and A. Bousquet‐Melou contributed to pharmacokinetic analysis and manuscript preparation.

### Peer Review

The peer review history for this article is available at https://publons.com/publon/10.1111/evj.13406.

## Data Availability

The data that support the findings of this study are available from the corresponding author upon reasonable request.
